# Supplementary material for: Inositol treatment inhibits medulloblastoma through suppression of epigenetic-driven metabolic adaptation
Source: Nat Commun. 2021 Apr 12;12:2148. doi: 10.1038/s41467-021-22379-7 (PMC8042111; doi:10.1038/s41467-021-22379-7)
Supplement: Supplementary file 1 — Supplementary Information [file 41467_2021_22379_MOESM1_ESM.pdf]

Supplementary Information

Inositol treatment inhibits medulloblastoma through suppression of epigenetic-driven metabolic adaptation.

Sara Badodi, Nicola Pomella, Xinyu Zhang, Gabriel Rosser, John Whittingham, Maria Victoria Niklison-Chirou, Yau Mun Lim, Sebastian Brandner, Gillian Morrison, Steven M. Pollard, Christopher D. Bennett, Steven C. Clifford, Andrew Peet, M. Albert Basson and Silvia Marino.

List of Supplementary Tables

Supplementary Table 1. List of primers

List of Supplementary Figures

Supplementary Figure 1. BMI1^{High};CHD7^{Low} signature increases EOMES expression and favours UBC lineage.

Supplementary Figure 2. BMI1^{High};CHD7^{Low} impacts FLT3, MET and mTOR pathway activation in MB cells, but not in hNSC.

Supplementary Figure 3. Impaired mitochondrial function in BMI1^{High};CHD7^{Low} MB cells, but not in hNSC with the same signature.

Supplementary Figure 4. BMI1-mediated epigenetic regulation of inositol-related pathways.

Supplementary Figure 5. IP6 cooperates with cisplatin in BMI1^{High};CHD7^{Low} MB xenografts.

Target gene	FW Primer sequence	REV Primer sequence
ACT B	5’-GCGAGAAGATGACCCAGATC-3’	5’-CCAGTGGTACGGCCAGAGG-3’
ATP5F1B	5’-CCCAGGCTGGTTCAGAGGT-3’	5’-AGGGGCAGGGTCAGTCAAG-3’
BMI1	5’-GCTGGTTGCCCATTGACAG-3’	5’-CGATGCATTTCTGCTTGATAA-3’
CHD7	5’-GAAGAAGATATAGAGACCCCAC-3’	5’-TCTTTGGTACATAACTTGGC-3’
FLT3	5’- CAATGATTCATCAGTGGGGA-3’	5’- TAAAGACCCAGAGACAGGAA-3’
MET	5’- TGAAGTATCAGCTTCCCAAC-3’	5’- CCAAGGAAAATGTGATGCTC-3’
PIP5K1B	5’- TCCTTATACGTCAATGAGCA-3’	5’- TTCCAAGGTAAATGTTGAACTG-3’

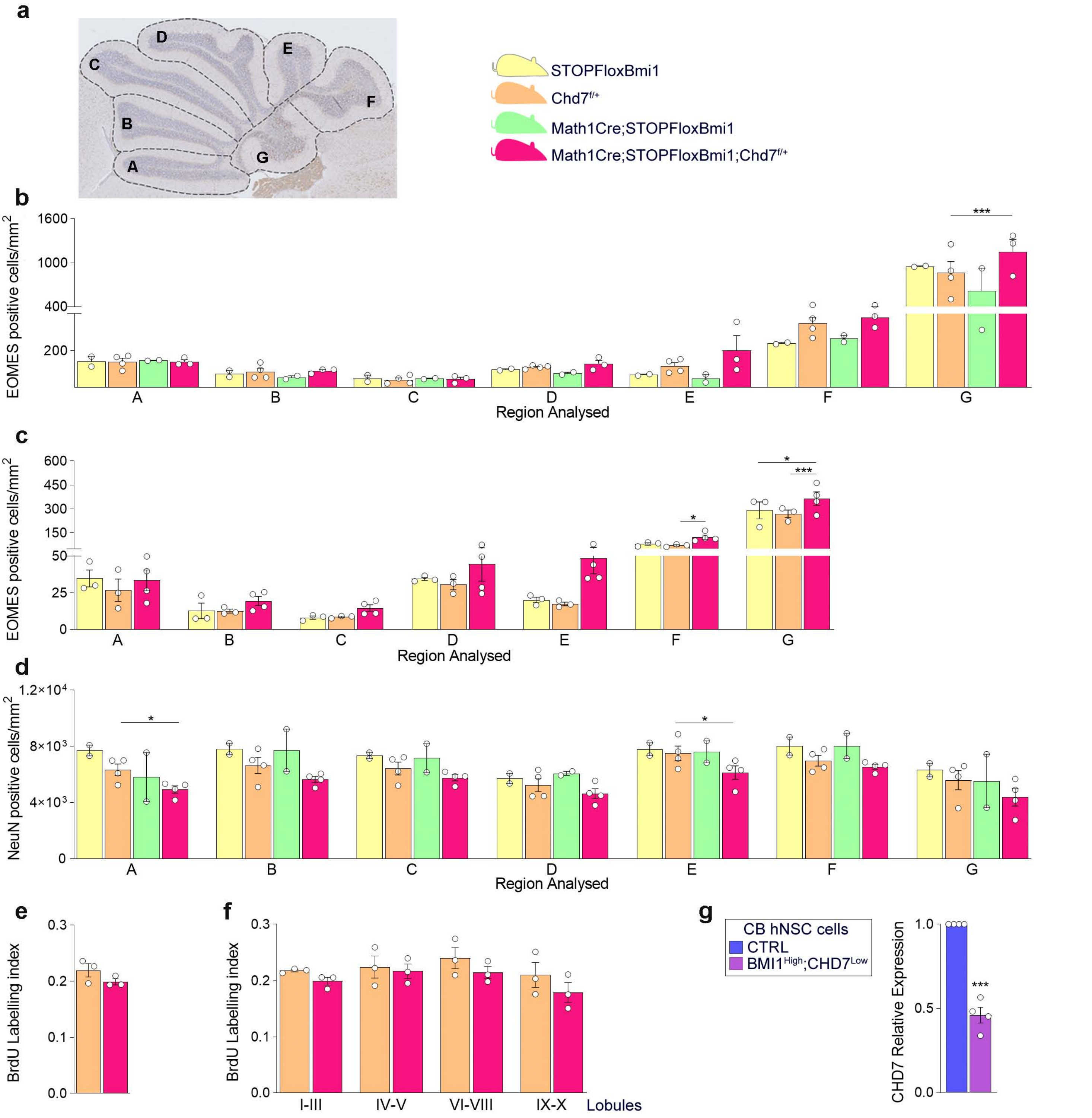
a
b
c
d
e
f
g
STOPFloxBmi1
Chd7f/+
Math1Cre;STOPFloxBmi1
Math1Cre;STOPFloxBmi1;Chd7f/+
EOMES positive cells/mm2
NeuN positive cells/mm2
Region Analysed
BrdU Labelling index
I-III
IV-V
VI-VIII
IX-X
Lobules
CB hNSC cells
CTRL
BMI1High;CHD7Low
CHD7 Relative Expression

Figure S1. BMI1^{High};CHD7^{Low} signature increases EOMES expression and favours UBC lineage.

a. Colours identify STOPFloxBmi1 (yellow), Chd7f/+ (orange), Math1Cre;STOPFloxBmi1 (green) or Math1Cre;STOPFloxBmi1;Chd7f/+ (pink) genotypes throughout the figure. Scheme of cerebellar regions identified and analysed in b, c and d.
b-c. EOMES^{+} UBC quantification in the cerebellar lobules at P7 (b) or P21 (c). (b) n=2 biological independent animals per STOPFloxBmi1 and Math1Cre;STOPFloxBmi1 genotypes, n=3 biological independent animals per Chd7$^{f/+}$ and Math1Cre;STOPFloxBmi1;Chd7$^{f/+}$ genotypes. (c) n=3 biological independent animals per STOPFloxBmi1 and Chd7$^{f/+}$ genotypes, n=4 biological independent animals per Math1Cre;STOPFloxBmi1;Chd7$^{f/+}$ genotype., two-way ANOVA.
d. NeuN^{+} GC quantification in cerebellar lobules at P7. n2 biological independent animals per STOPFloxBmi1 and Math1Cre;STOPFloxBmi1 genotypes, n=3 biological independent animals per Chd7$^{f/+}$genotype, n=4 biological independent animals per Math1Cre;STOPFloxBmi1;Chd7$^{f/+}$ genotype, two-way ANOVA.
e-f. Quantification of GCp proliferation in P7 EGL by BrdU incorporation in the whole cerebellum (f) or in the different lobules (f). n=3 biological independent animals per genotype.
g. qPCR analysis of *CHD7* expression in hNSC upon CHD7 silencing (BMI1^{High};CHD7^{Low}, purple) compared to control (CTRL, violet). n=4 biological independent experiments, two-tailed unpaired *t*-test.
All graphs report mean ± SEM. P values: *p<0.05 or ***p<0.001.
Source data are provided as a Source Data file.

Figure S2 Badodi et al.

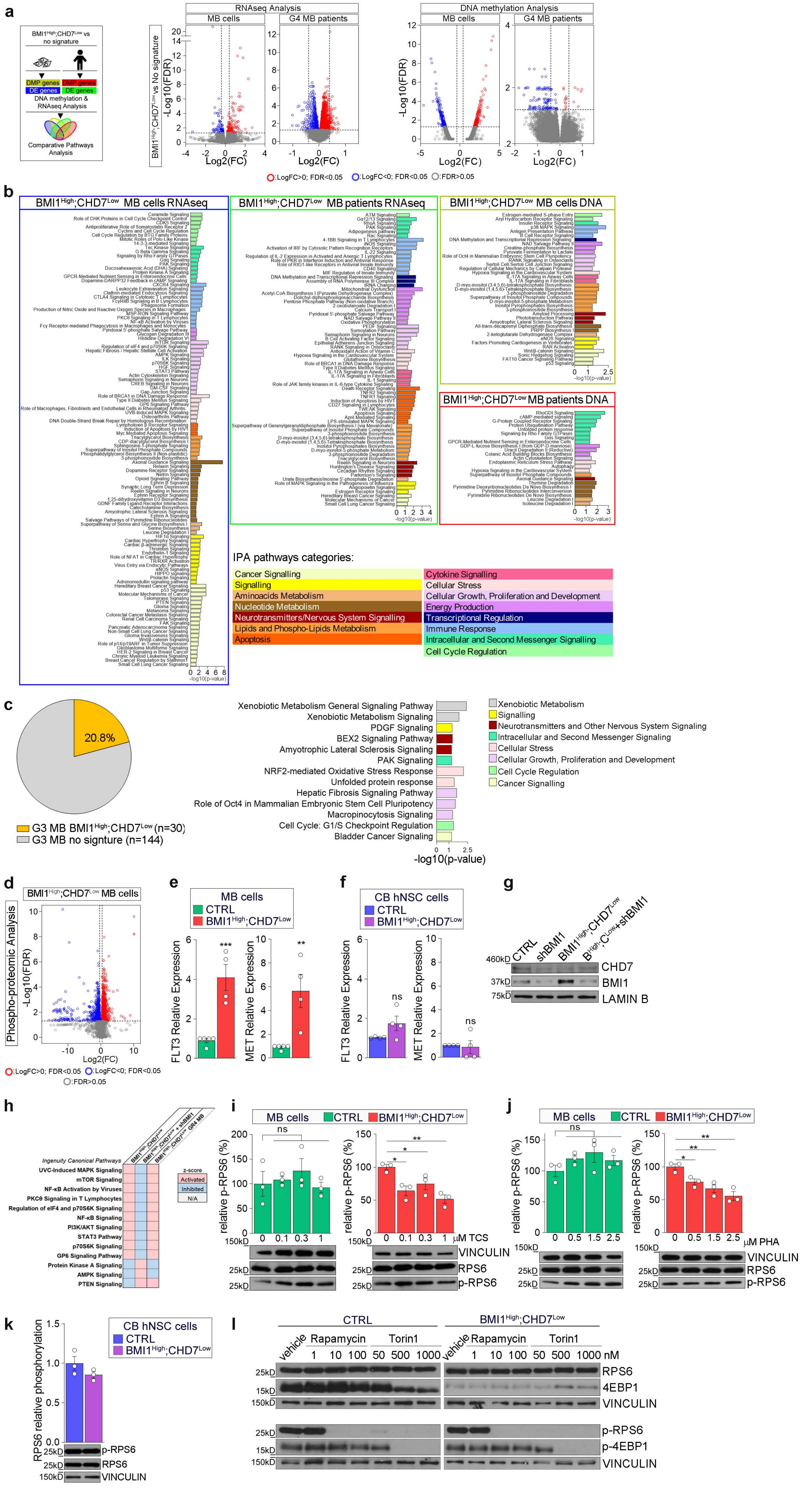

Figure S2. BMI1^{High};CHD7^{Low} impacts FLT3, MET and mTOR pathway activation in MB cells, but not in hNSC.

a. Schematic representation of the comparative analysis performed and volcano plots of genes differentially methylated or expressed between samples with and without signature. Red and blue dots represent genes with $Log_2FC > 0$ or < 0 and FDR<0.05 respectively, genes not significantly regulated (FDR>0.05) are represented in grey.
b. Histograms showing -log10(FDR) values of canonical pathways differentially enriched between MB cells or G4 patients with or without BMI1^{High};CHD7^{Low} signature identified in RNA-Seq or DNA methylation analysis. Canonical pathways are classified based on IPA categories list and colour coded accordingly.
c. Pie chart representing percentages of G3 MB tumour samples with (yellow) or without (grey) BMI1^{High};CHD7^{Low} signature (left panel). Histograms showing -log10(FDR) values of canonical pathways differentially enriched between G3 patients with or without BMI1^{High};CHD7^{Low} signature identified in RNA-Seq analysis. Canonical pathways are classified based on IPA categories list and colour coded accordingly (right panel).
d. Volcano plot of phospho-proteins differentially regulated in BMI1^{High};CHD7^{Low} MB cells compared to control. Red and blue dots represent phospho-peptides with $Log_2FC > 0$ or < 0 and FDR<0.05 respectively, phospho-peptides not significantly regulated (FDR>0.05) are represented in grey.
e-f. qPCR analysis showing quantification of *FLT3* and *MET* expression in MB cells (e) or hNSC (f) with (BMI1^{High};CHD7^{Low}) or without the signature (CTRL). n=4 biological independent experiments, two-tailed unpaired *t*-test.
g. Western blot analysis of BMI1 and CHD7 expression in nuclear extracts of the MB cells used in Figure 2i. LAMIN B immunoreactivity was used to normalize nuclear protein loading. Representative blot of n=3 biological independent experiments.
h. Heatmap representing significantly enriched (activated in red and inhibited in blue) mTOR upstream and downstream pathways in the comparison between MB cells with or without the signature. Activation or inhibition (z-scores) of the same pathways upon concomitant BMI1 silencing or in G4 MB tumour samples with the same signature are also represented.
i-j. Western blot and quantification of phosphorylated/total RPS6 (pRPS6, Ser240/244) after 72 hours treatment with increasing concentrations of FLT3 (TCS) or MET (PHA) inhibitors in control (green) or BMI1^{High};CHD7^{Low} (red) MB cells. VINCULIN immunoreactivity was used to normalize protein loading. n=3 biological independent experiments, two-way ANOVA.
k. Western blot and quantification of phosphorylated/total RPS6 (pRPS6, Ser240/244) in hNSC with or without the signature. VINCULIN immunoreactivity was used to normalize protein loading. n=3 biological independent experiments.
l. Western blot of phosphorylated RPS6 (p-RPS6, Ser240/244) and 4EBP1 (p-4EBP1, Thr37/46) upon treatment with increasing concentration of Rapamycin and Torin. VINCULIN immunoreactivity was used to normalize protein loading. Representative blot of n=3 biological independent experiments.
All graphs report mean ± SEM.P-values: *p<0.05, **p<0.01 or ***p<0.001.
Source data are provided as a Source Data file.

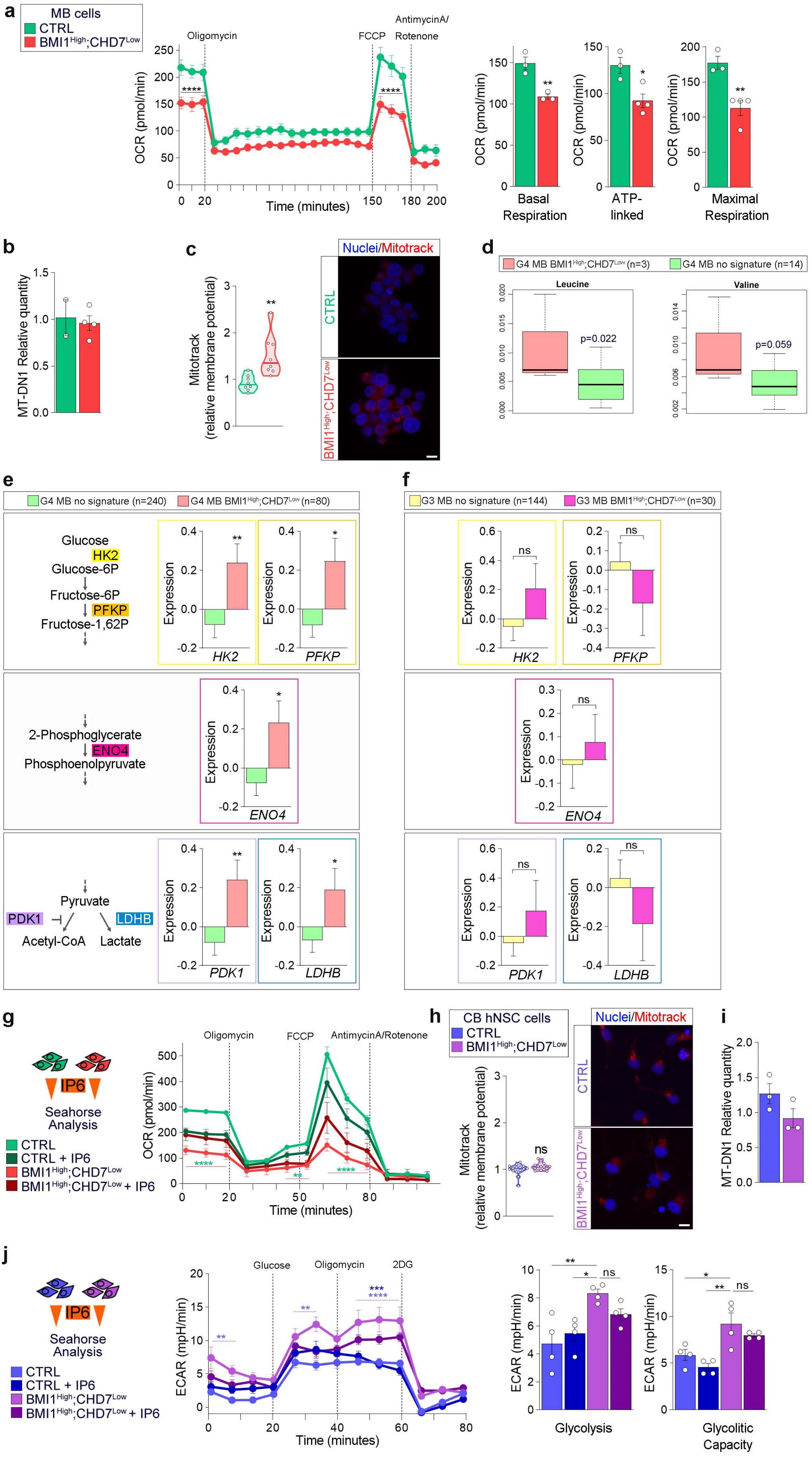
a
MB cells
CTRL
BMI1High;CHD7Low
Oligomycin
FCCP
AntimycinA/ Rotenone
OCR (pmol/min)
Time (minutes)
****
Basal Respiration
ATP-linked
Maximal Respiration
b
MT-DN1 Relative quantity
c
Mitotrack (relative membrane potential)
**
Nuclei/Mitotrack
CTRL
BMI1High;CHD7Low
d
G4 MB BMI1High;CHD7Low (n=3)
G4 MB no signature (n=14)
Leucine
p=0.022
Valine
p=0.059
e
G4 MB no signature (n=240)
G4 MB BMI1High;CHD7Low (n=80)
Glucose
HK2
Glucose-6P
Fructose-6P
PFKP
Fructose-1,62P
2-Phosphoglycerate
ENO4
Phosphoenolpyruvate
Pyruvate
PDK1
LDHB
Acetyl-CoA
Lactate
Expression
f
G3 MB no signature (n=144)
G3 MB BMI1High;CHD7Low (n=30)
ns
g
IP6
Seahorse Analysis
CTRL
CTRL + IP6
BMI1High;CHD7Low
BMI1High;CHD7Low + IP6
Oligomycin
FCCP
AntimycinA/Rotenone
OCR (pmol/min)
Time (minutes)
h
CB hNSC cells
CTRL
BMI1High;CHD7Low
Mitotrack (relative membrane potential)
ns
Nuclei/Mitotrack
i
MT-DN1 Relative quantity
j
IP6
Seahorse Analysis
CTRL
CTRL + IP6
BMI1High;CHD7Low
BMI1High;CHD7Low + IP6
Glucose
Oligomycin
2DG
ECAR (mpH/min)
Time (minutes)
Glycolysis
Glycolitic Capacity

Figure S3. Impaired mitochondrial function in BMI1^{High};CHD7^{Low} MB cells, but not in hNSC with the same signature.

a. Analysis of Oxidative Consumption Rate (OCR) using seahorse extracellular flux assay. BMI1^{High};CHD7^{Low} (red) or control (green) ICb1299 MB cells were sequentially treated with oligomycin, P-trifluoromethoxy carbonyl cyanide phenylhydrazone (FCCP) or a combination of antimycin A and rotenone at the indicated time points (dashed lines). Histograms (right) show OCR production during basal, ATP-linked and maximal respiration. n=3 biological independent experiments, two-tailed unpaired *t*-test.
b. qPCR quantification of *MT-DN1* expression in BMI1^{High};CHD7^{Low} or control MB cells. n=2 biological independent experiments per CTRL or n=4 biological independent experiments per BMI1^{High};CHD7^{Low}.
c. Quantification (left) and representative immunofluorescence analysis (right) of BMI1^{High};CHD7^{Low} or control MB cells stained with MitoTracker Red CMXRos (red), the accumulation of which is dependent on mitochondrial membrane potential. Nuclei are stained in blue. n=8 biological independent experiments, two-tailed unpaired *t*-test.
d. Ex-vivo MRS quantification of leucine and valine quantity comparing BMI1^{High};CHD7^{Low} (n=3) or G4 MB tissues without the signature (n=14). The boxplots represent median with interquartile ranges, two-tailed unpaired *t*-test.
e. Expression (z-score transformed) of genes coding for *HK2* (Hexokinase 2), *PFKP* (Phosphofructokinase 1), *ENO4* (Enolase 4), *PDK1* (Pyruvate Dehydrogenase Kinase 1) and *LDHB* (Lactate Dehydrogenase B) in G4 MB tumour samples with (n=80) or without (n=240) BMI1^{High};CHD7^{Low} signature. Two-tailed unpaired *t*-test. Diagrams represent steps of glucose metabolism regulated by the analysed enzymes.
f. Expression (z-score transformed) of genes coding for *HK2*, *PFKP*, *ENO4*, *PDK1* and *LDHB* in G3 MB tumour samples with (n=30) or without (n=144) BMI1^{High};CHD7^{Low} signature, two-tailed unpaired *t*-test.
g. Analysis of Oxidative Consumption Rate (OCR) using seahorse extracellular flux assay. BMI1^{High};CHD7^{Low} or control MB cells were incubated for 24 hours with IP6 1mM and then sequentially treated with oligomycin, P-trifluoromethoxy carbonyl cyanide phenylhydrazone (FCCP) or a combination of antimycin A and rotenone at the indicated time points (dashed lines). n=4 biological independent experiments per CTRL or n=5 per CTRL+IP6, BMI1^{High};CHD7^{Low} and BMI1^{High};CHD7^{Low}+IP6, one-way ANOVA.
h. Quantification (left) and representative immunofluorescence analysis (right) of BMI1^{High};CHD7^{Low} (purple) or control (violet) hNSC cells stained with MitoTracker Red CMXRos (red), the accumulation of which is dependent on mitochondrial membrane potential. Nuclei are stained in blue. n=15 biological independent experiments.
i. qPCR quantification of *MT-DN1* expression in BMI1^{High};CHD7^{Low} or control hNSC cells. n=3 biological independent experiments.
j. Analysis of ExtraCellular Acidification Rate (ECAR) using seahorse extracellular flux assay. BMI1^{High};CHD7^{Low} or control hNSC were incubated for 24 hours with IP6 1mM and then sequentially treated with glucose, oligomycin or 2-Deoxy-d-glucose (2DG) at the indicated time points (dashed lines). Histograms (right) show ECAR representing glycolysis and glycolytic capacity. n=4 biological independent experiments, one-way ANOVA.
All graphs report mean ± SEM. P-value: *p<0.05, **p<0.01, ***p<0.001 or ****p<0.0001. Scale bars=10µm.
Source data are provided as a Source Data file.

Figure S4

Badodi et al.

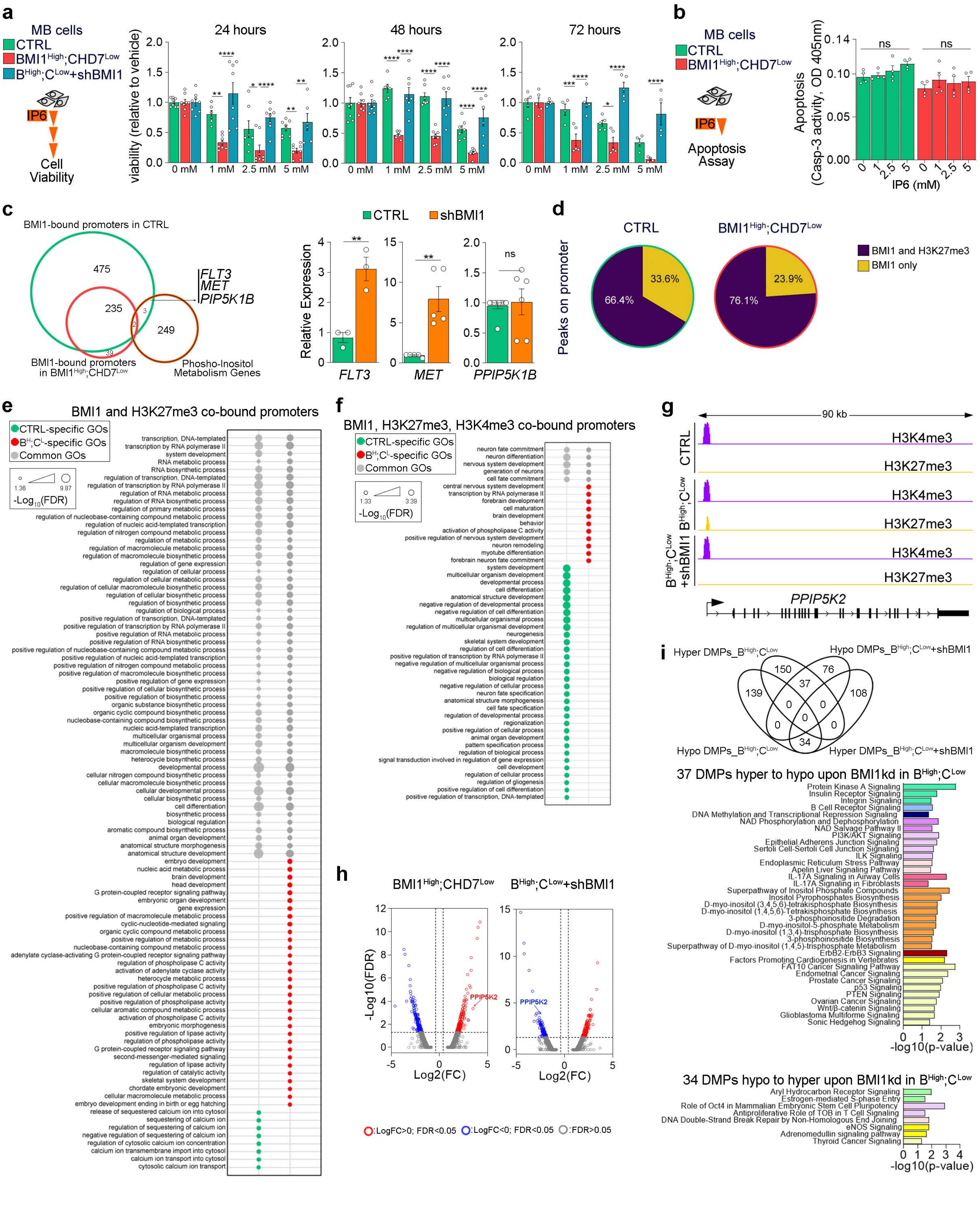

Figure S4. BMI1-mediated epigenetic regulation of inositol-related pathways.

a. Cell viability assays of control (green), $BMI1^{High}$;$CHD7^{Low}$ (red) or $BMI1^{High}$;$CHD7^{Low}$ with concomitant BMI1 silencing (B^{High};C^{Low}+shBMI1, blue) MB cells upon 24, 48 or 72 hours treatment with increasing concentrations of IP6. Histograms represent percentages of viable cells relative to non-treated cells. n=6 biological independent experiments, two-way ANOVA.
b. Apoptosis analysis of control or $BMI1^{High}$;$CHD7^{Low}$ MB cells as assessed by activated Caspase-3 activity. n=4 biological independent experiments, two-way ANOVA.
c. Venn diagram showing overlaps between genes bound by BMI1 on promoter in control (green) or $BMI1^{High}$;$CHD7^{Low}$ (red) MB cells and genes involved in phospho-inositol compounds metabolism (orange). Highlighted name of overlapping genes (left). qPCR analysis of *FLT3* (n=3 biological independent experiments), *MET* (n=5 biological independent experiments), and *PPIP5K1B* (n=6 biological independent experiments) in control (green) or BMI1 silenced (orange) MB cells (right). Two-tailed unpaired *t*-test.
d. Pie charts showing fraction of BMI1-bound genes with promoter occupied by BMI1 only (gold) or BMI1 and H3K27me3 (dark violet) in control or $BMI1^{High}$;$CHD7^{Low}$ MB cells.
e. Biological process GO terms enrichment for genes with promoter co-bound by BMI1 and H3K27me3. In green terms present only in control, in red $BMI1^{High}$;$CHD7^{Low}$-specific and common terms in grey. Size of the bubble represent -log10(FDR).
f. Biological process GO terms enrichment for genes with promoter co-bound by BMI1, H3K27me3 and H3K4me3. In green terms present only in control, in red $BMI1^{High}$;$CHD7^{Low}$-specific and common terms in grey. Size of the bubble represent -log10(FDR).
g. Genome browser view showing H3K4me3 (violet) and H3K27me3 (yellow) ChIP-Seq peaks around *PPIP5K2* promoter in control, $BMI1^{High}$;$CHD7^{Low}$ (B^{High};C^{Low}) or $BMI1^{High}$;$CHD7^{Low}$ with concomitant silencing of BMI1(B^{High};C^{Low}+shBMI1) MB cells.
h. Volcano plots of genes with differentially methylated probes between $BMI1^{High}$;$CHD7^{Low}$ vs CTRL (left) or B^{High};C^{Low}+shBMI1 vs $BMI1^{High}$;$CHD7^{Low}$ (right). Red and blue dots represent respectively genes with Log_2FC >0 or <0 and FDR<0.05, genes not significantly regulated (FDR>0.05) are represented in grey. Probe on PPIP5K2 is highlighted.
i. Venn diagrams showing overlap between significant probes represented in g. Pathway analysis of overlapping gene lists with histogram showing -log10(FDR) values of canonical pathways enriched.
All graphs report mean ± SEM. P values: *p<0.05, **p<0.01, ***p<0.001 or ****p<0.0001.
Source data are provided as a Source Data file.

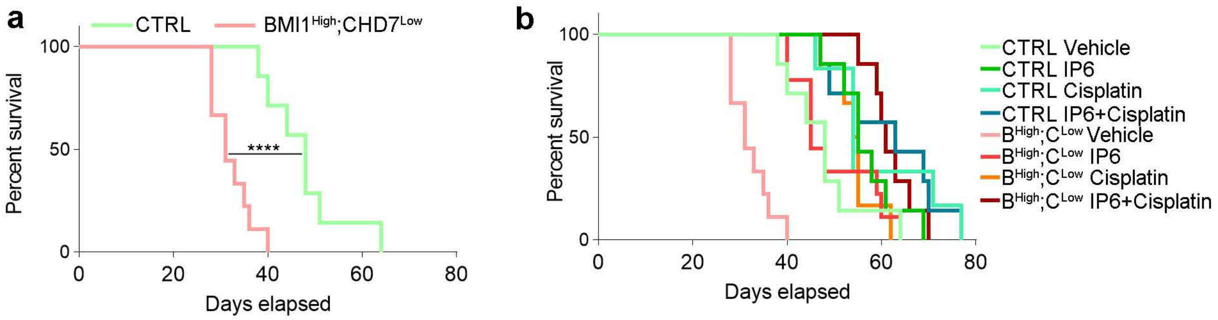

c

		CTRL				$B^{High};C^{Low}$			
		Vehicle	IP6	CISP	IP6+CISP	Vehicle	IP6	CISP	IP6+CISP
CTRL	Vehicle		ns	0.0323	0.0285	0.0003	ns	ns	0.0219
	IP6	ns		ns	ns	0.000067	ns	ns	ns
	CISP	0.0323	ns		ns	0.000187	ns	ns	ns
	IP6+CISP	0.0285	ns	ns		0.000067	0.028409	ns	ns
$B^{High};C^{Low}$	Vehicle	0.0003	0.000067	0.000187	0.000067		0.000034	0.0002	0.000067
	IP6	ns	ns	ns	0.028409	0.000034		ns	0.0284
	CISP	ns	ns	ns	ns	0.0002	ns		0.0217
	IP6+CISP	0.0219	ns	ns	ns	0.000067	0.0284	0.0217	

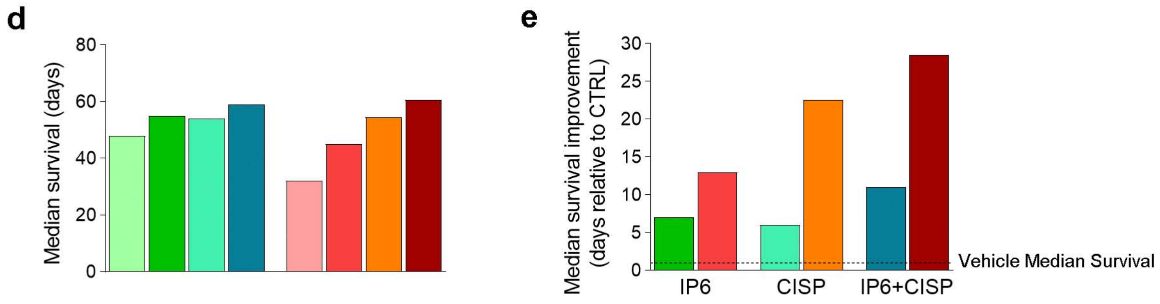

Figure S5. IP6 cooperates with cisplatin in $BMI1^{High}$;$CHD7^{Low}$ MB xenografts.

a. Kaplan-Meier survival curves of mice orthotopically xenografted with $BMI1^{High}$;$CHD7^{Low}$ (light red) or control (light green) MB cells. n=7 biological independent animals per CTRL group, n=9 biological independent animals per $BMI1^{High}$;$CHD7^{Low}$ group, two-tailed p-value determined by log-rank test.

b. Kaplan-Meier survival curves of mice orthotopically xenografted with $BMI1^{High}$;$CHD7^{Low}$ or control MB cells treated with vehicle, IP, Cisplatin or both (IP6+Cisplatin). n=6 biological independent animals per Cisplatin treated groups, n=7 biological independent animals per IP6+Cisplatin treated groups, n=8 biological independent animals per CTRL IP6 treated group, n=9 biological independent animals per $BMI1^{High}$;$CHD7^{Low}$ IP6 treated group, n=7 biological independent animals per CTRL Vehicle treated group, n=9 biological independent animals per $BMI1^{High}$;$CHD7^{Low}$ Vehicle treated group.

c. Table reporting two-tailed p-values of Kaplan-Meier survival curves in Figure S5b determined by log-rank test.

d-e. Histograms showing median survival day (d) of mice treated as in b and median survival improvement (e) calculated as the difference in days between median survival obtain with or without a specific treatment. Vehicle median survival equal to 1.

P value: ****$p<0.0001$.

Source data are provided as a Source Data file.
