# Supplementary material for: Inositol treatment inhibits medulloblastoma through suppression of epigenetic-driven metabolic adaptation
Source: Nat Commun. 2021 Apr 12;12:2148. doi: 10.1038/s41467-021-22379-7 (PMC8042111; doi:10.1038/s41467-021-22379-7)
Supplement: Supplementary file 3 — Reporting Summary [file 41467_2021_22379_MOESM3_ESM.pdf]

nature research

Corresponding author(s): Silvia Marino

Last updated by author(s): Mar 1, 2021

# Reporting Summary

Nature Research wishes to improve the reproducibility of the work that we publish. This form provides structure for consistency and transparency in reporting. For further information on Nature Research policies, see our Editorial Policies and the Editorial Policy Checklist.

## Statistics

For all statistical analyses, confirm that the following items are present in the figure legend, table legend, main text, or Methods section.

| n/a | Confirmed | |
|---|---|---|
| ☐ | ☒ | The exact sample size (*n*) for each experimental group/condition, given as a discrete number and unit of measurement |
| ☐ | ☒ | A statement on whether measurements were taken from distinct samples or whether the same sample was measured repeatedly |
| ☐ | ☒ | The statistical test(s) used AND whether they are one- or two-sided<br>*Only common tests should be described solely by name; describe more complex techniques in the Methods section.* |
| ☐ | ☒ | A description of all covariates tested |
| ☐ | ☒ | A description of any assumptions or corrections, such as tests of normality and adjustment for multiple comparisons |
| ☐ | ☒ | A full description of the statistical parameters including central tendency (e.g. means) or other basic estimates (e.g. regression coefficient) AND variation (e.g. standard deviation) or associated estimates of uncertainty (e.g. confidence intervals) |
| ☐ | ☒ | For null hypothesis testing, the test statistic (e.g. *F*, *t*, *r*) with confidence intervals, effect sizes, degrees of freedom and *P* value noted<br>*Give P values as exact values whenever suitable.* |
| ☒ | ☐ | For Bayesian analysis, information on the choice of priors and Markov chain Monte Carlo settings |
| ☒ | ☐ | For hierarchical and complex designs, identification of the appropriate level for tests and full reporting of outcomes |
| ☒ | ☐ | Estimates of effect sizes (e.g. Cohen's *d*, Pearson's *r*), indicating how they were calculated |

*Our web collection on statistics for biologists contains articles on many of the points above.*

## Software and code

Policy information about availability of computer code

| | |
|---|---|
| Data collection | StepOne Software v2.3 (ThermoFisher), ImageJ 1.8.0 (NIH), Excell 2016 (Office) |
| Data analysis | StepOne Software v2.3 (ThermoFisher), ImageJ 1.8.0, Trimgalore v0.6.5, STAR v2.6.1, Bioconductor packages (NOISeq, biomaRt, edgeR, GenomicRanges, ChIPSeeker, ChAMP), R software v3.5.3, Bowtie v2.3.4, Python v2.7.15, MACS2 algorithm, deeptools algorithms (bamCompare, bigWigCompare and plotHeatmap), Ingenuity Pathway Analysis (IPA, Qiagen), Reactome, Panther Gene Ontology tool, Cytoscape v. 3.7.2, Mascot Distiller (version 2.7.1), Pescal Software, GraphPad Prism 8, Combenefit 2.02 |

For manuscripts utilizing custom algorithms or software that are central to the research but not yet described in published literature, software must be made available to editors and reviewers. We strongly encourage code deposition in a community repository (e.g. GitHub). See the Nature Research guidelines for submitting code & software for further information.

## Data

Policy information about availability of data

All manuscripts must include a data availability statement. This statement should provide the following information, where applicable:

- Accession codes, unique identifiers, or web links for publicly available datasets
- A list of figures that have associated raw data
- A description of any restrictions on data availability

The authors declare that all the data supporting the findings of this study are available within the Article and its Supplementary Information files. The datasets generated in this study and processed data are available in the NCBI Gene Expression Omnibus database (GSE156077, https://www.ncbi.nlm.nih.gov/geo/query/acc.cgi?acc=GSE156077) or are available from the corresponding author upon reasonable request. Source data are provided with this paper.
Publicly available datasets used in the study: GSE85217 (Expression data from primary medulloblastoma samples), Reactome (https://reactome.org/), Panther Gene Ontology tool (http://geneontology.org/), Swiss Prot (https://www.uniprot.org/statistics/Swiss-Prot), PhosphoSite (https://www.phosphosite.org/), Phospho.ELM

(http://phospho.elm.eu.org/), PhosphoPOINT (http://kinase. bioinformatics.tw/).

# Field-specific reporting

Please select the one below that is the best fit for your research. If you are not sure, read the appropriate sections before making your selection.

☒ Life sciences ☐ Behavioural & social sciences ☐ Ecological, evolutionary & environmental sciences

For a reference copy of the document with all sections, see nature.com/documents/nr-reporting-summary-flat.pdf

# Life sciences study design

All studies must disclose on these points even when the disclosure is negative.

| | |
|---|---|
| Sample size | Required sample sizes were calculated by an a priori power analysis (alpha=0.05 and 1-beta=0.8). |
| Data exclusions | No data were excluded |
| Replication | Experiments were repeated with biological replica (n). Number of n specified for each experiments in Figure Legend. |
| Randomization | Gender-matched mice were randomly allocated to each experimental arm and randomly assigned to treatment groups. |
| Blinding | All experiments, when relevant, were performed blinded. Quantification of IHC staining was assessed by automated quantification without prior knowledge of sample details. Non blinded analysis were conducted for in vitro treatments requiring multiple round of adminstrations because the same drugs combination needed to be administrated in the correct sample. |

# Reporting for specific materials, systems and methods

We require information from authors about some types of materials, experimental systems and methods used in many studies. Here, indicate whether each material, system or method listed is relevant to your study. If you are not sure if a list item applies to your research, read the appropriate section before selecting a response.

## Materials & experimental systems

| n/a | Involved in the study | |
|---|---|---|
| ☐ | ☒ | Antibodies |
| ☐ | ☒ | Eukaryotic cell lines |
| ☒ | ☐ | Palaeontology and archaeology |
| ☐ | ☒ | Animals and other organisms |
| ☒ | ☐ | Human research participants |
| ☒ | ☐ | Clinical data |
| ☒ | ☐ | Dual use research of concern |

## Methods

| n/a | Involved in the study | |
|---|---|---|
| ☐ | ☒ | ChIP-seq |
| ☒ | ☐ | Flow cytometry |
| ☒ | ☐ | MRI-based neuroimaging |

## Antibodies

Antibodies used

The following primary antibodies were used: mouse monoclonal anti-BMI1 (1:1000, clone AF27, Active Motif), anti-GAPDH (1:1000, G8795, Sigma) and anti-Vinculin (1:5000, V4505, Sigma); rabbit monoclonal anti-phospho-RPS6 (Ser240/244, 1:1000, D68F8, Cell Signaling), anti- 4EBP1 (1:1000, 53H11, Cell Signaling), anti-phospho-4EBP1 (Thr37/46, 1:1000, 236B4, Cell Signaling); rabbit polyclonal anti-CHD7 (1:500, ab117522, abcam), anti-RPS6 (1:1000, 5G10, Cell Signaling), anti-PPIP5K2 (1:500, 16836, Novus Biological) and goat polyclonal anti-Lamin B (1:5000, sc-6216, Santa Cruz).
The following dye was used: 0.02 nM MitoTracker Red CMXRos (THermoFisher Scientific).
For ChIPseq analysis 4µg of antibody against BMI1 (BMI1, clone AF27, Active Motif), CHD7 (ab117522, abcam), H3K27me3 (pAb-195-050, Diagenode) or H3K4me3 (pAb-003-050, Diagenode) were used.

Validation

All antibodies used in the study are validated by the manufacturers. Further references to published studies that used of the antibody are provided on online datasheet on manufacturers web site.
https://www.merckmillipore.com/GB/en/product/Anti-BMI1-Antibody-clone-AF27,MM_NF-05-1321?ReferrerURL=https%3A%2F%2Fwww.google.com%2F&bd=1
https://www.sigmaaldrich.com/catalog/product/sigma/g8795?lang=en&region=GB
https://www.sigmaaldrich.com/catalog/product/sigma/v4505?lang=en&region=GB
https://www.cellsignal.com/products/primary-antibodies/phospho-s6-ribosomal-protein-ser240-244-d68f8-xp-rabbit-mab/5364
https://www.cellsignal.com/products/primary-antibodies/4e-bp1-53h11-rabbit-mab/9644
https://www.cellsignal.com/products/primary-antibodies/phospho-4e-bp1-thr37-46-236b4-rabbit-mab/2855
https://www.abcam.com/chd7-antibody-ab117522.html

https://www.cellsignal.com/products/primary-antibodies/s6-ribosomal-protein-5g10-rabbit-mab/2217
https://www.novusbio.com/products/hisppd1-antibody_nbp2-16836
https://www.abcam.com/products?sortOptions=Relevance&&selected.classification=Primary+antibodies&keywords=Laminin&gclid=Cj0KCQiAhP2BBhDdARIsAJEzXlFRwjSQCUrKsm6FXPF7-_KxiUVIFHIE-BrtSl2kQ1_-YSL1kdqwjZMaAsrZEALw_wcB&gclsrc=aw.ds
https://www.diagenode.com/en/p/h3k27me3-polyclonal-antibody-premium-50-mg-27-ml
https://www.diagenode.com/en/p/h3k4me3-polyclonal-antibody-premium-50-ug-50-ul

## Eukaryotic cell lines

Policy information about cell lines

| | |
|---|---|
| Cell line source(s) | ICb1299 patient-derived MB lines were obtained from Dr Xiao-Nan Li, Baylor College of Medicine, Texas Children Cancer Centre, USA . CHLA-01-Med MB cells were purchased from ATCC (CRL3021). Human foetal NSC lines were obtained from the Cancer Research UK-funded Glioma Cellular Genetics Resource (www.gcgr.org.uk). |
| Authentication | Cells used were commercially available or obtained from cell banks performing in house authentication. Regular and periodic morphology check and collection of morphology images for comparison. |
| Mycoplasma contamination | Cells tested negative. |
| Commonly misidentified lines (See ICLAC register) | No commonly misidentified cell lines were used in the study. |

## Animals and other organisms

Policy information about studies involving animals; ARRIVE guidelines recommended for reporting animal research

| | |
|---|---|
| Laboratory animals | Male and female NOD SCID mice of 3 weeks were used. Orthotopic injections were performed with P5 mice. |
| Wild animals | No wild animals were used in the study. |
| Field-collected samples | No field-collected samples were used in the study. |
| Ethics oversight | All procedures were performed in accordance with licenses held under the UK Animals (Scientific Procedures) Act 1986 and later modifications and conforming to all relevant guidelines and regulations. |

Note that full information on the approval of the study protocol must also be provided in the manuscript.

## ChIP-seq

### Data deposition

- [x] Confirm that both raw and final processed data have been deposited in a public database such as GEO.
- [x] Confirm that you have deposited or provided access to graph files (e.g. BED files) for the called peaks.

| | |
|---|---|
| Data access links<br>*May remain private before publication.* | The datasets generated in this study and processed data are available in the NCBI Gene Expression Omnibus database (GSE156077, https://www.ncbi.nlm.nih.gov/geo/query/acc.cgi?acc=GSE156077) |
| Files in database submission | 1299_SCR antiBMI1, 1299_SCR antiH3K4me3, 1299_SCR antiH3K27me3, 1299_SCR Input<br>1299_1_SCR antiBMI1, 1299_1_SCR antiH3K4me3, 1299_1_SCR antiH3K27me3, 1299_1_SCR Input<br>1299_shC antiBMI1, 1299_shC antiH3K4me3, 1299_shC antiH3K27me3, 1299_shC Input<br>1299_1_shC antiBMI1, 1299_1_shC antiH3K4me3, 1299_1_shC antiH3K27me3, 1299_1_shC Input<br>3021_SCR antiBMI1, 3021_SCR antiH3K4me3, 3021_SCR antiH3K27me3, 3021_SCR Input<br>3021_1_SCR antiBMI1, 3021_1_SCR antiH3K4me3, 3021_1_SCR antiH3K27me3, 3021_1_SCR Input<br>3021_shC antiBMI1, 3021_shC antiH3K4me3, 3021_shC antiH3K27me3, 3021_shC Input<br>3021_1_shC antiBMI1, 3021_1_shC antiH3K4me3, 3021_1_shC antiH3K27me3, 3021_1_shC Input |
| Genome browser session (e.g. UCSC) | no longer applicable |

### Methodology

| | |
|---|---|
| Replicates | Four independent biological replica obtained from two different cell lines were analysed. |
| Sequencing depth | The average Phred score of the surviving reads across all samples was 30 and the average sequencing depth was 36.3 M (min = 22.1 M, max = 56.7 M). |

| | |
|---|---|
| Antibodies | For ChIPseq analysis 4µg of antibody against BMI1 (BMI1, clone AF27, Active Motif), CHD7 (ab117522, abcam), H3K27me3 (pAb-195-050, Diagenode) or H3K4me3 (pAb-003-050, Diagenode) were used. |
| Peak calling parameters | Peaks were called via the MACS2 algorithm (subroutine callpeak) against the corresponding input background. The shifting model was disabled to make different datasets comparable and the "--broad" option was enabled for the analysis of the histone mark H3K27me3. |
| Data quality | After performing post-alignment quality checks, peaks were called via the MACS2 algorithm (subroutine callpeak) against the corresponding input background. A minimum fold enrichment of 2 was selected with an FDR of 0.05, in both narrow peak and broad peak (--broad-cutoff) statistical analyses. |
| Software | The quality of ChIP-Seq samples was first assessed via FastQC and TrimGalore. The alignment to the Ensembl GRCh38 human reference genome was performed via Bowtie v2.3.4. The exploratory tools deeptools, plotCorrelation, plotPCA and plotFingerprint on Python v2.7.15 were used to further assess sample characteristics. Peaks were called via the MACS2 algorithm (subroutine callpeak). The Bioconductor packages in R GenomicRanges and ChIPSeeker were used to find regions of consensus peaks between the two cell lines, for each antibody/condition, and to annotate them based on the location with respect to the nearest transcription start site (TSS). The versions of all relevant Bioconductor packages were compatible with R v3.5.3.<br>The deeptools algorithms bamCompare, bigWigCompare and plotHeatmap were used to produce relevant bigwig files and heatmaps. |
